# Supplementary material for: Methylglyoxal-Derived Advanced Glycation End Product (AGE4)-Induced Apoptosis Leads to Mitochondrial Dysfunction and Endoplasmic Reticulum Stress through the RAGE/JNK Pathway in Kidney Cells
Source: Int J Mol Sci. 2021 Jun 18;22(12):6530. doi: 10.3390/ijms22126530 (PMC8235496; doi:10.3390/ijms22126530)
Supplement: Supplementary file 1 [file ijms-22-06530-s001.zip › ijms-1240456-supplementary.pdf]

Table S1. Primer sequences for quantitative RT-PCR

Gene	Accession no.	Primer sequence 5'-3'	
		**Forward**	**Reverse**
**RAGE (Human)**	NM_001136.5	AAA CAT CAC AGC CCG GAT TG	TCC GGC CTG TGT TCA GTT TC
**RAGE (Mouse)**	XM_006523501.4	TTG GAG AGC CAC TTG TGC TA	CCC TCA TCG ACA ATT CCA GT
**Cyclin B1 (Human)**	NM_031966.4	CTGCTGGGTGTAGGTCCTTG	TGCCATGTTGATCTTCGCCT
**Cyclin B1 (Mouse)**	NM_172301.3	AAGGTGCCTGTGTGTGAACC	GTCAGCCCCATCATCTGCG
**XBP1 (Human)**	NM_005080.4	CCAAGGGGAATGAAGTGAGGC	GCTGGCAGGCTCTGGGGAAG
**CHOP (Human)**	NM_001195053.1	CAG AAC CAG CAG AGG TCA CA	AGC TGT GCC ACT TTC CTT TC
**CHOP (Mouse)**	XM_030244867.1	TAT CTC ATC CCC AGG AAA CG	GGG CAC TGA CCA CTC TGT TT
**ATF4 (Human)**	XM_017028807.2	CCA ACA ACA GCA AGG AGG AT	GGG GCA AAG AGA TCA CAA GT
**ATF4 (Mouse)**	NM_009716.3	GAG CTT CCT GAA CAG CGA AGT G	TGG CCA CCT CCA GAT AGT CAT C
**GRP78 (Human)**	NM_005347.5	AGT GGT GCC TAC CAA GAA GTC TCA	TGT CAG GGG TCT TTC ACC TTC ATA
**GRP78 (Mouse)**	NM_001163434.	GAA AGG ATG GTT AAT GAT GCT GAG	GTC TTC AAT GTC CGC ATC CTG
**Bax (Human)**	NM_001291428.2	TCA GGA TGC GTC CAC CAA GAA G	TGT GTC CAC GGC AAT CAT C
**Bax (Mouse)**	XM_011250780.4	TTC CGA GTG GCA GCT GAG ATG TTT	TGC TGG CAA AGT AGA AGA GGG CAA
**Bcl-2 (Human)**	NM_000633.3	ATG TGT GTG GAG AGC GTC AAC C	TGA GCA GAG TCT TCA GAG ACA GCC
**Bcl-2 (Mouse)**	NM_009741.5	GAG TAC CTG AAC CGG CAT CTG	CTT CAG AGA CAG CCA GGA GAA A
**p53 (Human))**	NM_000546.6	GCG AGC ACT GCC CAA CAA CA	GGA GAC ATC GTC TGG GGT GT
**p53 (Mouse**	KF766124.1	TGA TGG AGA GTA TTT CA CC	GGG CAT CCT TTA ACT CTA AG
**KIM-1 (Human)**	AW604297	TCACCTCACCACCACAAACCC	CAC CCA AAA CAC CAA CAA CCA C
**GAPDH (Human)**	AP023475.1	GCT AAG CAG TTG GTG GTG CA	TCA CCA CCA TGG AGA AGG C
**GAPDH (Mouse)**	XM_036165840.1	CCA ATG TGT CCG TCG TGG ATC T	GTT GAA GTC GCA GGA GAC AAC C

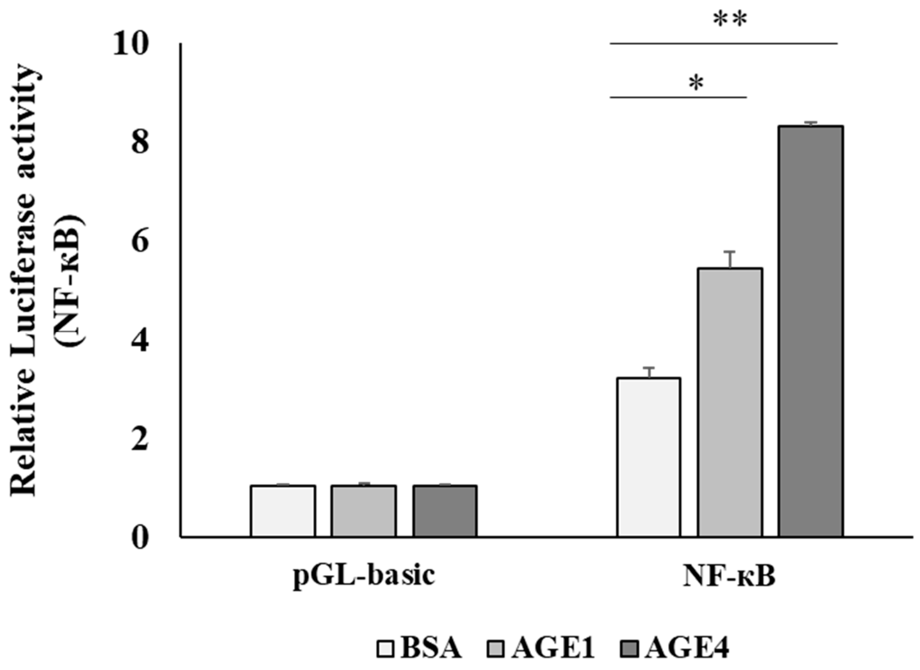

Figure S1. The cultured HEK 293 cells were transfected with NF-κB promoter driven luciferase reporter plasmid DNA. Cells were harvested and measured for luciferase reporter activity. The columns showed the relative luciferase NF-κB promoter activity. HEK293 cells transfected with NF-κB promoter driven luciferase reporter gene were pre-treated with AGEs (200 μg/mL for 24 h). The luciferase activity was identified using Dual-Luciferase® Reporter 1000 Assay System kit. Data are expressed as the mean ± standard deviation (SD) of three independent experiments. (*$p < 0.05$, **$p < 0.01$)

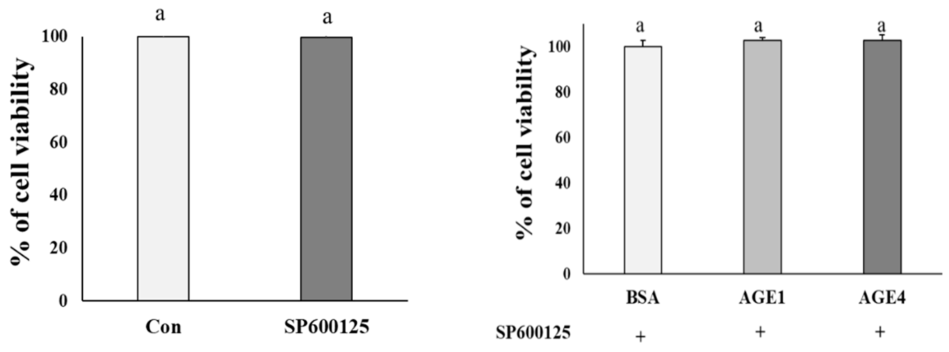

Figure S2. Effect of JNK pathway inhibitor, SP600125 in kidney cells that have been treated or not treated with AGEs. The cell survival rates are measured using MTT analysis. (a) The cell was incubated with SP600125 10 μM for 24 h (b) The cell was treated with 10 μM SP600125 for 3 h, washed once with PBS, and treated with 200 μg/mL of AGEs for 24 h. Data are expressed as the mean ± standard deviation (SD) of three independent experiments ($p < 0.05$).

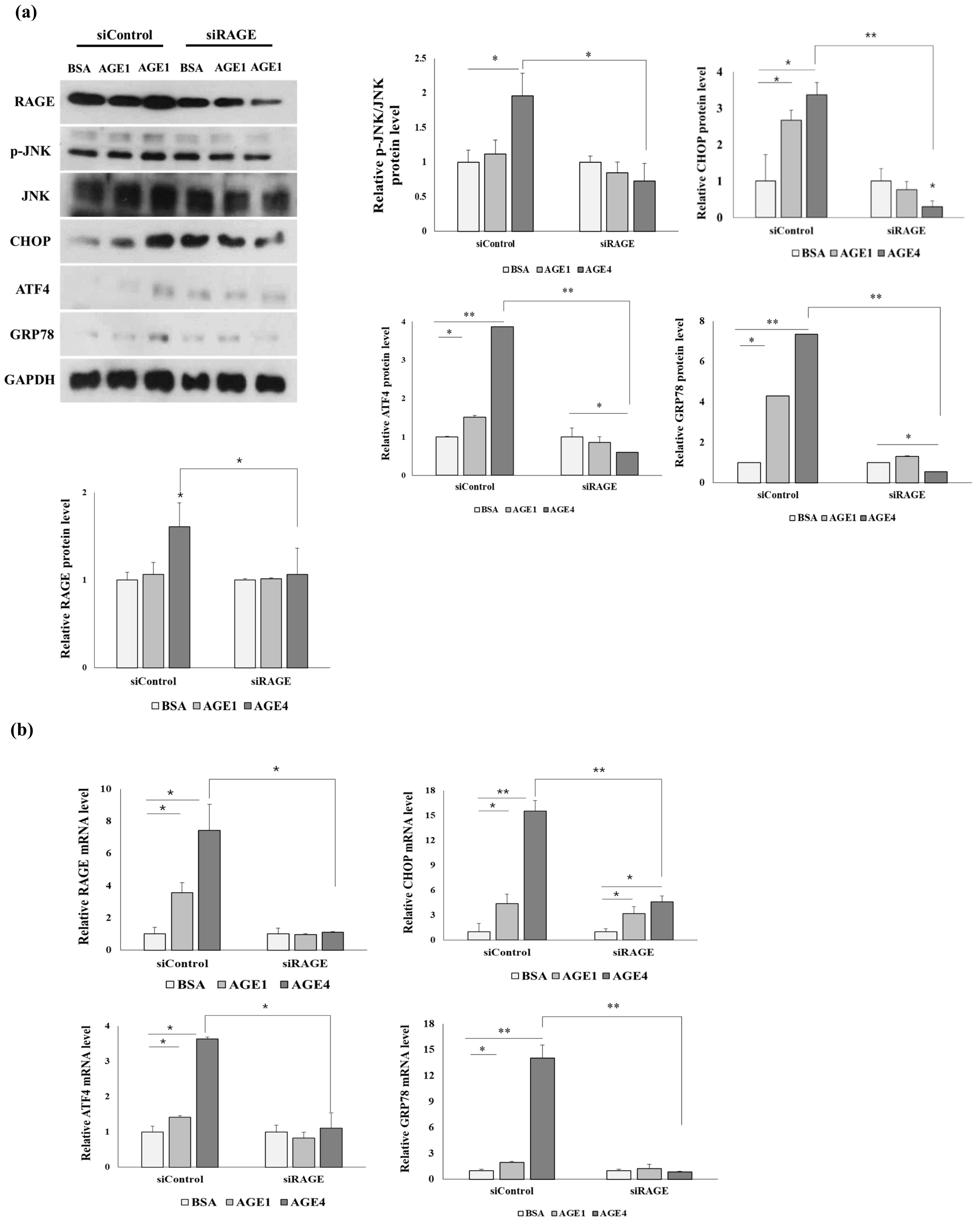
(a)
siControl
siRAGE
BSA AGE1 AGE1 BSA AGE1 AGE1
RAGE
p-JNK
JNK
CHOP
ATF4
GRP78
GAPDH
Relative p-JNK/JNK protein level
Relative CHOP protein level
Relative ATF4 protein level
Relative GRP78 protein level
Relative RAGE protein level
siControl
siRAGE
BSA AGE1 AGE4
(b)
Relative RAGE mRNA level
Relative CHOP mRNA level
Relative ATF4 mRNA level
Relative GRP78 mRNA level
siControl
siRAGE
BSA AGE1 AGE4

Figure S3. AGE4-induced ER stress dependent RAGE. Before treatment of the AGEs, cells were transfected with the RAGE small double stranded interfering RNAs (siRNA) silencing or siControl by using lipofectamine 2000. (a)The protein and (b) mRNA expression of siRAGE were detected by western blot and q-RT-PCR. The protein fold used Image J software to quantify the GAPDH into a graph. Data are expressed as the mean ± standard deviation (SD) of three independent experiments. *$p < 0.05$, and **$p<0.01$ siRAGE compared with the siControl by *t*-test.

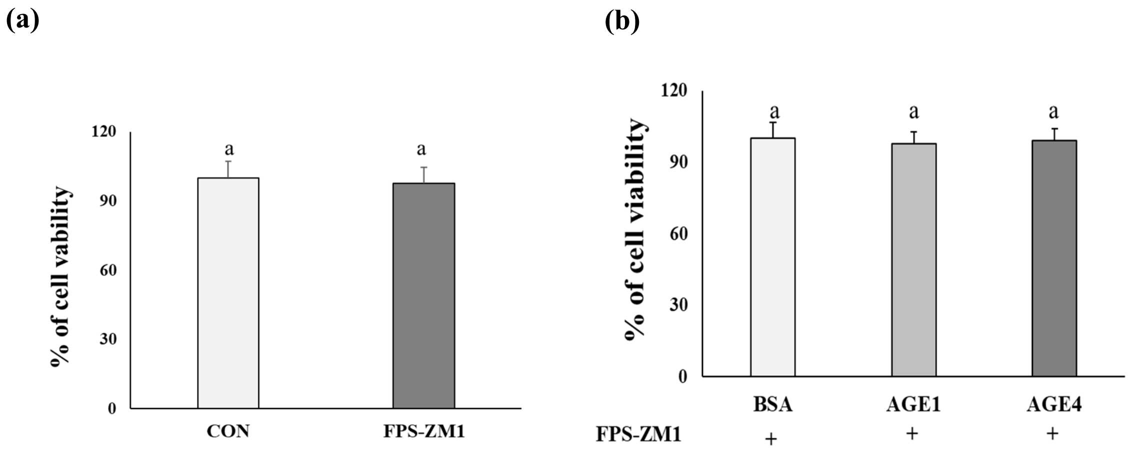

Figure S4. Effect of RAGE inhibitor in kidney cells that have been treated or not treated with AGEs. The cell survival rates are measured using MTT analysis. (a) The cell was incubated with FPS-ZM1 10 μM for 24 h. (b) The cell was treated with 10 μM FPS-ZM1 for 3 h, washed once with PBS, and treated with 200 μg/mL of AGEs for 24 h. Data are expressed as the mean ± standard deviation (SD) of three independent experiments (p< 0.05).

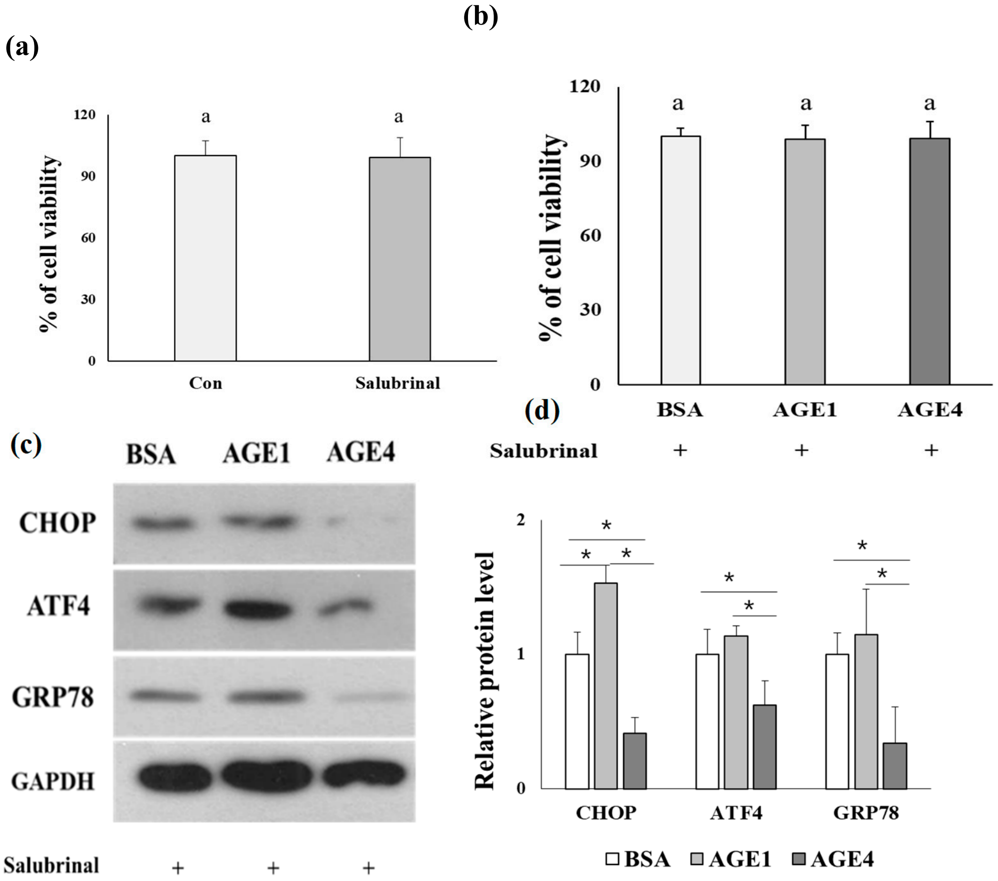

Figure S5. Effect of ER stress inhibitor in kidney cells that have been treated or not treated with AGEs. The cell viability rates are measured using MTT analysis. (a) The cell was incubated with salubrinal 10 μM for 24 h (b) The cell was treated with salubrinal 10 μM for 3 h, washed once with PBS, and treated with 200 μg/mL of AGEs for 24 h. (c) The induction of CHOP, ATF4, and GRP78 protein by AGE4 was decreased with salubrinal pretreatment. Data are expressed as the mean ± standard deviation (SD) of three independent experiments (p<0.05).

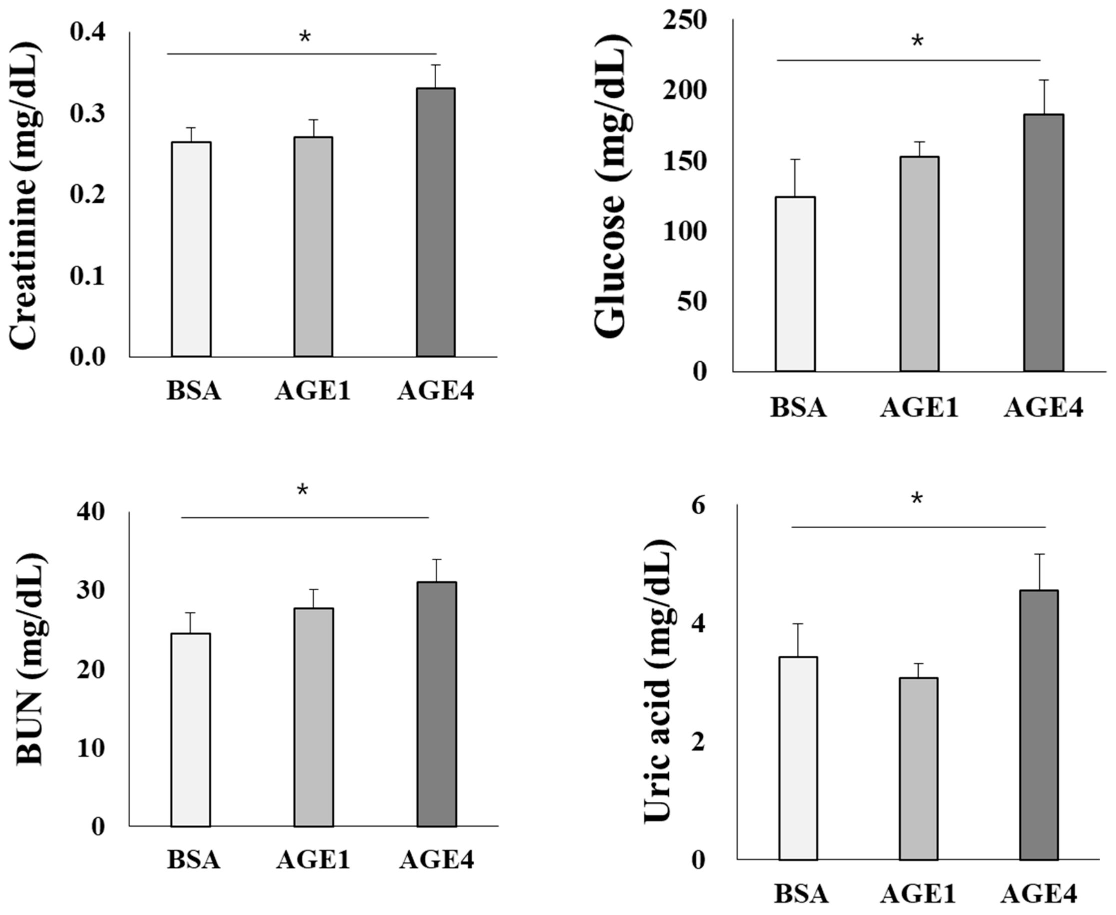

Figure S6. Effect of AGE4 on the blood serum analysis on mice blood. AGE4 administration regulates creatinine, glucose, BUN, and uric acid levels in C57/BL6N mice. The mice were orally administered 800 mg/kg/bw for three weeks. The levels of creatinine, glucose, BUN, and uric acid were greater in the AGE4-fed group than in BSA-fed group. Data are expressed as the mean ± standard deviation (SD) (n=4, p<0.05 compared each group by t-test).
